# Gender Differences in Peer Influence on Autistic Traits in Special Needs Schools—Evidence From Staff Reports

**DOI:** 10.3389/fpsyg.2021.718726

**Published:** 2021-11-04

**Authors:** Gina Nenniger, Verena Hofmann, Christoph M. Müller

**Affiliations:** Department of Special Education, University of Fribourg, Fribourg, Switzerland

**Keywords:** autistic traits, autism spectrum disorder, peer influence, gender, intellectual disabilities, special needs schools

## Abstract

Children and adolescents with an intellectual disability (ID) and autistic traits often attend special needs schools where they are surrounded by peers with diverse characteristics. Given the role that peers can play in social development, we examined whether autistic traits development in students with ID and high levels of such characteristics are influenced by the level of autistic traits among the schoolmates they like most. Furthermore, we investigated the degree to which this peer influence susceptibility depends on students’ gender. A longitudinal design, with data collection points at the beginning and the end of a school year, was used. Staff reported on 330 students with high levels of autistic traits (20.6% girls; age 10.17 years, *SD* = 3.74) who attended 142 classrooms in 16 Swiss special needs schools. Results showed that students’ future individual level of autistic traits (T2) was not predicted by the autistic traits level of preferred peers (T1), controlling for individual autistic traits at T1, level of general functioning, gender, and age. However, the peer effect was significantly moderated by students’ gender, indicating that girls but not boys were susceptible to peer influence. These findings are discussed in terms of implications for understanding autistic traits development and directions of support for children and adolescents in their peer context.

## Introduction

Autism spectrum disorder (ASD) is characterized by difficulties in social communication, social interaction, and repetitive behaviors and interests (American Psychiatric Association, 2013) that contribute to impairments in multiple domains of social development. ASD is often associated with an intellectual disability (ID), defined by intellectual and adaptive functioning about two standard deviations below the general population (American Psychiatric Association, 2013). About 33–70% of children and adolescents with ASD also have an ID (Ritvo et al., 1989; Fombonne, 2005; Charman et al., 2011; Knopf, 2020) and about 8–39% of individuals with ID are estimated to have ASD, depending on diagnostic criteria used, sampling procedures, and measurement instruments used (see, e.g., La Malfa et al., 2004; de Bildt et al., 2005; Tonnsen et al., 2016). However, it must be noted that several individuals with ID show certain autistic-like behaviors without necessarily having ASD. This is due to the fact that autistic-like behavior (e.g., restricted and repetitive behaviors) sometimes can also result from difficulties in intellectual and adaptive functioning (see, e.g., Bhaumik et al., 2010; Berry et al., 2018). Furthermore, individuals may exhibit only some behaviors described as symptoms of ASD, but not all. These types of behaviors in the literature are often termed “autistic traits” and they are considered as being on a continuum ranging from almost no autistic traits in individuals without an ASD diagnosis to high levels of autistic traits in all relevant dimensions, fulfilling the criteria for ASD (see, e.g., de Groot and van Strien, 2017). In this study we were specifically interested in students with ID and high levels of autistic traits. Compared to students without ID or those with ID but no or very low levels of autistic traits, those with ID and high levels of autistic traits tend to be specifically at risk for less advantageous outcomes (see, e.g., Matson and Shoemaker, 2009).

While ASD diagnosis is relatively stable throughout the life span (Woolfenden et al., 2012), the quantity and quality of autistic traits can change over time, and this variation is related to individual factors such as cognitive or verbal abilities (Seltzer et al., 2004; McGovern and Sigman, 2005; Shattuck et al., 2007). For example, individuals with an ASD diagnosis and additional ID were found to improve less over time in non-verbal communication and social reciprocity than those without ID (Shattuck et al., 2007). Additionally, contextual factors such as family characteristics (e.g., maternal praise, mother-child relationship) are associated with changes in the development of autistic traits (Greenberg et al., 2006; Shattuck et al., 2007; Woodman et al., 2015).

A contextual factor in autistic traits development that has been less studied is the influence of peers. Within the school context, students’ peers include schoolmates and classmates (Müller and Zurbriggen, 2016). Research on typically developing children and adolescents indicates peers from school can have considerable impact on individual behavioral development (Brown et al., 2008). According to social learning theories, several mechanisms underpin these influences, such as imitation and peers’ social reinforcement (Bandura and Walters, 1963; Brown et al., 2008; Akers, 2009). Thus, schools provide an important environment for social learning among children and adolescents. While peer influence research on behavioral development has primarily focused on students’ externalizing and internalizing behaviors (see, e.g., Brechwald and Prinstein, 2011), less is known regarding the degree to which peers from school also influence autistic traits development, especially with regard to students with ID.

Worldwide, students who exhibit high levels of autistic traits are taught in various school settings, such as inclusive or special needs classrooms. When associated with an ID, there is a specifically high probability that special needs settings are attended (Harris and Handleman, 2000; Dolev et al., 2014). This is also the case for Switzerland, where the present study was conducted. Eckert (2015) reported that about 25% of all students with an ASD diagnosis in Switzerland attended a special needs school for kindergarten, about 57% for primary school, and about 73% for upper secondary school. Since children and adolescents with high levels of autistic traits tend to experience limited social participation in their spare time (Shattuck et al., 2011; Ratcliff et al., 2018), peers from school are a particularly important peer group for these individuals.

Just as with typically developing students, children and adolescents with ID and high levels of autistic traits can be expected to interact preferentially with some peers but less with others (see also, Hall and Smith, 2009). Indeed, students with an ASD diagnosis were found to prefer interacting with individuals who hold common interests, others with ASD, and sometimes also with much younger or older individuals (Orsmond et al., 2004; American Psychiatric Association, 2013; Morrison et al., 2020). As those peers who are most liked can be expected to be most salient and wield the greatest influence over individual students, we were interested in understanding the degree to which preferred peers from school influence future autistic traits among students with ID and high levels of autistic traits. Furthermore, girls and boys with ASD are known to differ from each other in various domains of social behavior (e.g., social skills, social relationships, social motivation; Head et al., 2014; Sedgewick et al., 2016). These differences could result in boys and girls with high levels of autistic traits being differentially influenced by their peers from school.

Given the described gaps in research, the current study investigated peer influence on autistic traits among students with ID and high levels of autistic traits attending special needs schools and specifically the role of students’ gender herein. More knowledge on this issue may improve understanding of how the social context influences the development of autistic traits and could provide new avenues for peer-based support and interventions at school.

### Peer Influence and Autistic Traits

A large body of research has demonstrated the social development of children and adolescents is influenced by both non-self-selected and self-selected peers (Juvonen and Galván, 2008; Brechwald and Prinstein, 2011). According to Kindermann (2016), non-self-selected peers include, for example, all students from a self-contained classroom or a school. Self-selected peers, in contrast, refer to children and adolescents with whom individuals interact both voluntarily and frequently, such as friends. A great part of peer influence research focuses on the role of self-selected peers, showing that this group has considerable influence on students’ behavioral and competence development (see, e.g., Vitaro et al., 2000; Hojjat and Moyer, 2017).

Peer influence occurs through several mechanisms. According to social learning theories, new behaviors can be learned by observing and imitating peer models. Observing models can also lead to activation or inhibition of already known behaviors, for example, when observing a model’s behavior is rewarded by others (Bandura, 2007). Peer reinforcement may include verbal prompts or non-verbal behavior, such as laughter (Kiesner et al., 2000; Dishion and Tipsord, 2011). Another peer influence mechanism relates to the desire to belong to a group or to avoid behavioral uncertainty, which causes people to conform with others (Cialdini and Goldstein, 2004). In this process, social norms play an important role. Both descriptive norms (i.e., what most others do; e.g., the average behavioral level in a group) and injunctive norms (i.e., what others approve or disapprove of) can impact individual behavior (Cialdini et al., 1991).

Very little is known on peer influence on autistic traits. When extrapolating from the literature on typically developing children and adolescents, some of ASD’s behavioral characteristics may suggest that children and adolescents with high levels of autistic traits are less susceptible to peer influence compared to others. For example, students with an ASD diagnosis have fewer social contacts and reciprocal friendships than typically developing children and adolescents (Petry, 2018), which may generally limit opportunities for peer influence. Furthermore, social learning theory assumes that the behavior of peers must be recognized and processed adequately for learning to ensue (Bandura, 2007). Children and adolescents with high levels of autistic traits often have difficulties recognizing the emotions and intentions of their peers (Nuske et al., 2013). In addition, they often tend to have a detail-focused perception of the environment (Happé and Frith, 2006; Müller and Nussbeck, 2008). In social situations, these tendencies could lead to greater perception of details (e.g., a specific action of a peer) relative to integrating information regarding the global picture of a complex social situation (e.g., taking into account the presence and reactions of other peers). Hence, social cues and their related social reinforcement may not always be perceived as such, resulting in less peer influence. Lastly, students with high levels of autistic traits often have special interests and focus on rituals (American Psychiatric Association, 2013), which might attenuate the desire to belong to a group, as well as decrease adaptation of individual behavior to peers’ behavior in favor of preserving rituals and routines.

Some studies provide insights on general social influence in individuals with an ASD diagnosis (i.e., without ID). Bowler and Worley (1994) investigated social conformity among eight adults with ASD using Asch’s line judgment task (Asch, 1956). Participants were asked to compare the length of a given line with the length of three other lines, while people not personally known to participants intentionally gave wrong answers. Results showed no overall differences in the degree of conformity between the ASD group and two control groups of typically developing adults and of young adults with mild learning disabilities matched on verbal intelligence. Lazzaro et al. (2019) confirmed these results using a verbal memory task and did not find differences in conformity among young adults with and without ASD. However, using an adapted version of Asch’s line judgment task with children with ASD, Yafai et al. (2014) reported that participants with ASD conformed less to the opinion of others than typically developing children. Similar results were found in a study by Izuma et al. (2011). In a game-like experiment where adults with and without ASD were asked to donate money under two different conditions, once in the presence of an observer and once without observer, typically developing participants changed their donation behavior in the presence of an observer the behavior of individuals with ASD was insensitive to the presence of an observer. The authors concluded individuals with ASD have difficulties considering their own reputation from the perspective of others (Izuma et al., 2011).

While the above studies relate to social influence in general, only a few have explicitly focused on the influence of *peers*. van Hoorn et al. (2017) investigated the extent to which peer feedback influenced prosocial behavior in male adolescents with and without ASD. Participants were asked to play a computer-based public good game under several conditions, where they had to decide whether to give tokens to themselves or donate them to their group of four peers (a “public good pot”). In one condition, peers evaluated these decisions by providing “likes” (thumb up) or “dislikes” (empty space). Both male adolescents with and without ASD were found to be sensitive to peer feedback on their prosocial decisions (i.e., receiving likes if two or more tokens were donated to the group). However, within the total sample a higher level of autistic traits and higher intelligence were associated with less sensitivity to peer feedback on antisocial donation decisions (i.e., receiving likes if three or fewer tokens were donated to the group).

To our knowledge, only one study has examined autistic traits as the subject of peer influence. This explorative study investigated teachers’ observations of peer influence among students with ASD in a special needs school that was only attended by students with ASD and low levels of adaptive functioning. Teacher reports on observed peer influence susceptibility in 23 students with ASD in this school context indicated that autistic traits were influenced by other students with ASD, although not often and to varying degrees across autistic trait subtypes (Nenniger and Müller, 2020). Teachers’ descriptions of how peer influence on autistic traits occurred suggested that different mechanisms are at play, including imitation and social reinforcement. For example, it was reported that a student imitates the stomping, repetitive movements of peers. In terms of social reinforcement, one student was reported to repeatedly make eye contact with a peer who reacted to the eye contact with verbal noises (Nenniger and Müller, 2020).

In sum, the literature contains few and rather contradictory results on peer influence susceptibility in individuals with an ASD diagnoses, and only one explorative study has considered autistic traits as the subject of peer influence. This same study by Nenniger and Müller (2020) was also the only one to consider peer influence in a natural peer context (i.e., school), whereas the other studies were conducted under standardized laboratory conditions. No research considered longitudinal associations between peers’ and individual autistic traits, which would be important to draw conclusions on peer socialization effects (Kindermann, 2016).

### The Role of Gender in Peer Influence on Autistic Traits

To our knowledge, no studies have yet investigated whether gender affects individuals’ susceptibility to peer influence on autistic traits. However, evidence does exist on gender differences for other behavioral domains, making it worth to also consider this issue with regards to autistic traits. For example, boys have been found to be more susceptible to peer influence than girls in terms of externalizing behaviors (Erickson et al., 2000; Steinberg and Monahan, 2007; Parsai et al., 2009; Müller et al., 2016) while girls appear to be more susceptible to peer influence on internalizing behaviors (Stevens and Prinstein, 2005; van Zalk et al., 2010; Conway et al., 2011). These findings may be explained by differing degrees of salience for certain behaviors, when considering social comparisons among boys vs. girls, and different patterns of preferred social activities (e.g., Pellegrini et al., 2004; Goodwin, 2006; Dean et al., 2017).

Regarding the potential role of gender as a moderating factor in peer influence on autistic traits, several differences between boys and girls with high levels of autistic traits could be important, such as their social skills, social relationships, or social motivation. Head et al. (2014) provided some evidence to support this possibility. In their study, 50 children and adolescents with an ASD diagnosis and typical levels of intellectual functioning reported on the nature and understanding of their friendships. Results showed that girls with ASD had significantly closer, more empathic, and supportive friendships than boys with ASD. One explanation for these findings is provided by the Camouflaging hypothesis which states that individuals with ASD, but mainly females, adapt and imitate social skills and develop coping mechanisms to hide their social difficulties (Wing, 1981; Head et al., 2014; Mandy, 2019). In line with the direction of these findings, Sedgewick et al. (2016) found that girls with an ASD diagnosis and low to typical level of intellectual functioning demonstrated a similar friendship quality to non-autistic girls, while the friendship quality of boys with ASD and low to typical level of intellectual functioning was lower than those of boys without ASD and girls with or without ASD. Furthermore, in this study girls with ASD exhibited more motivation for social contact. These results suggest that boys and girls with high levels of autistic traits differ in their social competence, which could in turn affect their peer influence susceptibility. For example, higher social skills (e.g., perceiving and understanding others’ emotions) among girls with high levels of autistic traits may enable them to better recognize social reinforcement from peers, and thus they may be also more susceptible to peer influence than boys. Furthermore, greater social motivation in girls with high levels of autistic traits may contribute to greater willingness to camouflage autistic traits and instead adapt their behavior to the descriptive peer group norms so as to avoid rejection by peers. Although such gender differences in the susceptibility to peer influence on autistic traits appear plausible, this issue has not been directly investigated.

### The Current Study

This study used a longitudinal research design to examine two research questions. First, we investigated whether children and adolescents with ID and high levels of autistic traits, in the following termed *target students*, are influenced by the peers they like the most in their special needs school context. Studying this question is particularly important as students with ID and high levels of autistic traits are at risk for less advantageous outcomes (see, e.g., Matson and Shoemaker, 2009). Hence, more knowledge on the influence of the social context may help to better understand the behavioral development of this population and provide perspectives for support and interventions. We expected that the level of autistic traits exhibited by the peers most liked by the target students would positively predict the target students’ individual level of autistic traits over time (Hypothesis 1). To test this effect, we used a classic peer influence research framework by predicting individual level of autistic traits at T2 by the level of autistic traits exhibited by the most liked peers at T1, controlling for the individual level of autistic traits at T1 (Kindermann and Gest, 2009). As earlier research found gender and age differences in peer influence susceptibility among typically developing individuals (see, e.g., Erickson et al., 2000; Steinberg and Monahan, 2007; Conway et al., 2011), we controlled for these factors in our analyses. To provide information about peer influence considered independently of the students’ levels of general functioning, we used this factor as an additional control variable. Second, we investigated whether boys and girls with ID and high levels of autistic traits differ in their susceptibility to peer influence. Since boys and girls with high levels of autistic traits differ, for example, in terms of social skills, social relationships, or social motivation, gender seems like an especially relevant variable in the context of peer influence (see also, Verrier et al., 2020). Given the described differences in social competence between girls and boys with high levels of autistic traits, we expected that target girls are more susceptible to peer influence on autistic traits than target boys (Hypothesis 2).

## Materials and Methods

### Participants

We used data from a longitudinal research project in the German-speaking part of Switzerland that assessed peer influence in special needs schools for students with ID (Müller et al., 2020). In Switzerland only children and adolescents with a clinical diagnosis of ID can attend special needs schools for students with ID, therefore all study participants exhibited intellectual functioning in the range of an ID. Diagnosis of ID in Switzerland is based on ICD-10 criteria and typically established using an IQ test (IQ < 70) and a clinical rating of adaptive behavior (World Health Organization, 2016). The overall study sample included 1,125 children and adolescents (69% boys; out of 1,177 students in total) attending 179 classrooms (out of 182 classrooms total) from the 16 participating special needs schools, for a participation rate of 95.58%. Data were collected at the beginning and end of a school year (T1: August–October 2018, T2: April–June 2019).

For the research questions at hand, we analyzed data from a subsample of 330 target students with ID and high levels of autistic traits (above the autism cut-off score in the measurement instrument used, see below). These students were distributed over 144 classrooms and 16 schools. They were on average 10.17 years old (*SD* = 3.74), ranging from 4.17 to 18.58 years, and 20.6% were female. Descriptive information on the demographics and level of general functioning of the students as measured with the instruments described below is displayed in Table 1 (no information on clinical diagnoses was available). In addition, data were used that provided information on the peers reported to be most liked by the target students (*N* = 317 overall; see more information in “Results” section).

**TABLE 1:** Descriptive results of the main study variables (*N* = 330).

	*M*	*SD*	Observed range	%
Individual autistic traits at T1	0.81	0.25	0.53–1.65	
Individual autistic traits at T2	0.72	0.31	0.10–1.63	
Autistic traits of liked peers at T1	0.41	0.24	0.00–1.48	
Level of general functioning T1	2.52	4.73	0.00–39.00	
Female gender				20.6
Age in years	10.17	3.74	4.17–18.58	

*Autistic traits: from 0 = not true to 2 = very true or often true.*

Information on target students was reported by 203 school staff members, including 83.3% female participants, mean age 45.52 years (*SD* = 11.32) at T1. In terms of training levels, 45.4% were special needs teachers, while others had other teacher trainings or were therapists, social workers, pedagogical staff, or long-term trainees. At T1, staff reported to know the students they reported on for *M* = 12.57 months (*SD* = 13.28). Staff members had 3 weeks to complete the questionnaires and make additional observations in the meantime. On average, each staff member reported on 1.60 students (*SD* = 0.97, range = 1–6). In 79.4% of the cases staff members filled out the questionnaires for the same students at T1 and T2. Due to personnel fluctuation, in 10.9% of the cases different staff members filled out the questionnaires at T1 and T2 (for the remaining staff members no information was available).

### Measures

#### Individual Level of Autistic Traits

The German version of the Developmental Behavior Checklist Teacher Form (DBC-T; Brereton et al., 2002; Einfeld and Tonge, 2002; Einfeld et al., 2007) was used to assess students’ autistic traits at T1 and T2. Teachers were asked to rate the presence or absence of a broad spectrum of behaviors on a 3-point scale (0 = *not true*, 1 = *somewhat true or sometimes true*, 2 = *very true or often true*). The DBC-T was developed to assess the behavior of children and adolescents with intellectual and developmental disabilities. It consists of 94 items and provides a score for total problem behavior, as well as scores for six subscales in the domains of disruptive behavior, self-absorbed behaviors, communication disturbance, anxiety, social relating, and the category “other” comprised of all remaining items. The German version is based on the original DBC-T and shows adequate validity and reliability (Einfeld et al., 2007). The instrument’s norms are based on an Australian sample of 640 young people, age 4–18, with ID (IQ < 50).

Part of the DBC can be used as a screening instrument for ASD. The Autism Screening Algorithm (DBC-ASA) uses a subset of 29 out of the 94 items of the DBC. Brereton et al. (2002) evaluated the DBC-ASA in a sample of 180 children and adolescents who had an ASD diagnosis based on DSM-IV criteria (American Psychiatric Association, 2000) and 180 non-autistic or typically developing children and adolescents matched for age, gender, and IQ level. Results showed the DBC-ASA provides good differentiation between children and adolescents with and without ASD. Steinhausen and Metzke (2004) evaluated the German DBC-ASA in a German-speaking sample of 84 individuals with ASD and 84 participants with ID matched by gender, age, and disability level (assessed with a four-item disability rating). They came to a slightly different algorithm for the autism screening, termed DBC-ASAR1. This scale includes 40 items (e.g., avoids eye contact, aloof, in his/her own world, does not respond to others’ feelings, upset over changes in routine/environment, repeats same word/phrase over) and contains no further subscales. The DBC-ASAR1 obtained the best cut-off score at 21, leading to a sensitivity of 0.85 and a specificity of 0.61. The scale showed very good internal consistency (α = 0.93). Given we conducted the DBC-T in its German version, we opted to use the DBC-ASAR1 scale to determine the level of autistic traits of students and found adequate reliability (α = 0.81) in the present dataset. Higher values indicate more severe levels of autistic traits. The cut-off score of 21 was used to identify the 330 target students with high levels of autistic traits out of the total study sample of 1,125 participating students.

#### Autistic Traits Among the Most Liked Peers

The peers most liked by the target students were determined using peer nominations filled out by school staff at T1. Many students attending special needs schools for students with ID experience severe disability-related difficulties providing such information (e.g., limited reading and writing ability, difficulties understanding specific questions). Hence, peer relations were assessed from the staff perspective. Applying an approach used similarly in earlier studies (see, e.g., Harks and Hannover, 2020), staff reported on peer relations based on their everyday school life observations. Given that staff in special needs schools spend the whole day with their students (including lunch) and fostering peer relations is part of their job mandate, these insights provide important information when not being able to directly assess the peers’ perspective.

School staff were provided a list of all the students in the given school and then asked to nominate the students whom the target student appeared to like most (“*Who does this student like especially in school?”*). Staff could nominate as many students as they considered appropriate, an approach recommended in the literature on peer nominations (e.g., Cillessen and Marks, 2017). Following a procedure often used in peer influence research, the average level of autistic traits (i.e., DBC-ASAR1 mean values) among the members of the peer group (i.e., preferred peers) for each target student at T1 was then determined (see, e.g., Vitaro et al., 2000; Stevens and Prinstein, 2005). Hence, each target student had a context score of the most liked peers’ mean level of autistic traits at the beginning of the school year.

#### Demographics

School staff reported on students’ gender (male or female) and age in months.

#### Level of General Functioning

To estimate students’ individual levels of general functioning, staff members filled out a German version of the Adaptive Behavior Assessment System-3 for teachers (ABAS-3; Bienstein et al., 2018). Together with IQ (which was not collected in this study), the assessment of adaptive behavior (i.e., everyday life skills) and interpretation of results relative to those of same-aged peers is required to determine ID and its severity (see, e.g., American Psychiatric Association, 2013; Tassé et al., 2016). Adaptive behavior scores compared to a reference norm from the general population can therefore be considered an acceptable estimator of general functioning in this study. The German ABAS-3 is based on the original US version (Harrison and Oakland, 2015), which is often used in ID assessment. It consists of 174 items that describe specific skills, each rated for a student on a scale from 0 = *is not able* to 3 = *always/almost always*. The instrument assesses 9 skill areas that are combined to form a conceptual score (Communication, Functional Academics, Self-Direction), social score (Leisure, Social), and practical score (Self-Care, School Living, Community Use, and Health and Safety), as well as an overall General Adaptive Composite (GAC). Norms of the ABAS-3 are based on a sample of 1,896 persons from the general US population. The instrument is well evaluated and provides adequate validity and reliability (Harrison and Oakland, 2015). For the current analyses we used the percentile rank of the overall score of adaptive functioning (in the present data α = 0.99), indicating the level of functioning relative to age with higher values showing less impairment.

### Procedure

The KomPeers-research project was reviewed and accepted in terms of scientific and ethical procedures by the Institutional Research Commission of the Department of Special Education of the University of Fribourg. Recruitment of participating schools was based on written information regarding the study and personal meetings with school headmasters. The entire data collection used a coding system that ensured complete anonymity, and researchers never had access to the names of staff, students, or parents at any time. Prior to data collection, parents received a letter from the school informing them about the study and the anonymity guaranteed to them and their child. The letter emphasized that no medical diagnoses would be assessed, and that participation was voluntary. Parents were offered the opportunity to decline to participate, in which case staff did not fill out questionnaires for their child. The letter was written using plain language principles and was translated into the nine most frequently used languages in Switzerland. School staff were likewise informed about the study and could refuse participation.

### Statistical Analyses

Preliminary analyses included a description of the main characteristics of the target children and adolescents (including changes from T1 to T2) and their most liked peers. In addition, bivariate correlations of the associations between the key variables were run.

In our main analyses, we accounted for the hierarchical data structure (Raudenbush and Bryk, 2002). As in our study we relied on professionals’ reports on students’ competences and sometimes more than one staff member per classroom participated, data on individual students can be considered to be nested within raters (i.e., staff; *M* = 1.60 students per rater, range = 1–6). Raters in turn were nested within classrooms (*M* = 1.41 raters per classroom, range = 1–4). Such a data structure can result in underestimation of standard errors which might lead to false significant effects (Raudenbush and Bryk, 2002) because scores of individuals reported by the same raters are likely more similar than scores of individuals reported by different raters, as raters may show specific answer patterns. Also, due to a similar classroom context, raters within the same classroom can be more similar to each other relative to raters of other classrooms. To control for the nested data structure and to avoid biased results, we estimated multilevel models with three levels (i.e., Level 1: students; Level 2: raters; Level 3: classrooms). We used the software Mplus which accounts for unbalanced data due to missing values by applying full information maximum likelihood estimation. As we did not expect peer influence to vary between raters and classrooms, longitudinal random intercept models with fixed slopes were conducted using the software MPlus (Muthén and Muthén, 2017). First, an unconditional model was estimated to determine variances and intraclass correlations (Model 1). Second, we predicted individual autistic behavior of target students at T2 by the average level of autistic traits among the peers liked most by the target students at T1, controlling for T1 individual autistic traits only (Model 2). To test Hypothesis 1, we then predicted the individual level of autistic traits of target students at T2 by the average level of autistic traits among the peers liked most by the target students, controlling for target students’ individual level of autistic traits at T1, gender, age, and level of general functioning (Model 3; see also, Kindermann and Gest, 2009). To test Hypothesis 2, the interaction term between the level of autistic traits among the peers liked most by the target students and the target students’ gender was added (Model 4). We used an alpha level of 0.05 for all statistical tests.

## Results

### Preliminary Analyses

As defined by the selection criteria in the Participants section, the 330 target students exhibited high levels of autistic traits as indicated by a DBC-ASAR1-Score of 21 and above (sum of ratings across items). At T1 these students’ individual level of autistic traits scale mean score was 0.81 (*SD* = 0.25, range = 0.53–1.65) and at T2 it was 0.72 (*SD* = 0.31, range = 0.10–1.63). A dependent sample *t*-test showed that the decrease in autistic traits from T1 to T2 was statistically significant (*p* < 0.001). A repeated measures ANOVA indicated there was no difference in the decrease in autistic traits between girls and boys (*p* = 0.445). Target students showed on average low levels of general functioning with a mean percentile rank of 2.52 (*SD* = 4.73; range = 0.00–39.00).

For 215 target students, at least one peer was nominated as being especially liked. For the remaining target students, no peer was nominated, indicating staff members did not perceive that these students had schoolmates they especially liked. Target students with peers nominated did not differ significantly from those without in terms of gender (*p* = 0.670). In contrast, target students for whom at least one preferred peer was nominated showed lower levels of autistic traits (*p* < 0.001), higher levels of general functioning (*p* = 0.003), and they were older (*p* = 0.048) than target students without nominations. Due to the resulting missing values, the data set was reduced to the 215 target students for whom preferred peers were nominated. In total, staff members nominated 446 peers as liked most by these target students (including multiple nominations). A dependent sample *t*-test showed that these nominated peers exhibited significantly lower levels of autistic traits (*M* = 0.41, *SD* = 0.24, range = 0.00–1.48) than the target students (*M* = 0.76, *SD* = 0.23, *p* < 0.001). Of the total nominations, 65.47% were made for peers who attended the target students’ classroom and 34.53% were made for peers attending other classrooms in the same school. Target students had on average 2.07 (*SD* = 1.43, range = 1–11) peers they liked most. Excluding multiple nominations, 341 peers were nominated, of which 62.8% were boys. Nominees had a mean age of 11.07 years (*SD* = 3.58, range = 4.17–18.58) and their average percentile rank of functioning level was 8.43 (*SD* = 11.67, range = 0.00–91.00).

Table 2 shows the correlations between the study variables used in the main analyses. More individual autistic traits by target students at the beginning of the school year were strongly associated with more individual autistic traits at the end of the school year (*r* = 0.690, *p* < 0.001). Individual autistic traits at T1 and T2 were not correlated with the mean levels of autistic traits of the most liked peers. For the variable gender we did not find any significant correlation. Higher levels of general functioning at T1 by target students were associated with less autistic traits at T1 (*r* = −0.128, *p* = 0.029) and T2 (*r* = −0.165, *p* = 0.006), indicating the lower the level of general functioning, the more autistic traits students showed. Furthermore, small negative correlations were found between age and individual autistic traits at T1 (*r* = −0.177, *p* = 0.001) and T2 (*r* = −0.144, *p* = 0.011), suggesting that the older students were, the less autistic traits they showed. Age and the percentile rank of general functioning at T1 were negatively correlated (*r* = −0.136, *p* = 0.020), indicating that for older students there were reported lower levels of functioning relative to their age.

**TABLE 2:** Correlations between the main study variables (*N* = 330).

	1	2	3	4	5	6
1. Individual autistic traits at T1	−	0.690*	0.091	−0.128*	0.027	−0.177*
2. Individual autistic traits at T2		−	0.104	−0.165*	–0.019	−0.144*
3. Autistic traits of liked peers at T1			−	−0.013	–0.051	–0.124
4. Level of general functioning T1				−	0.006	−0.136*
5. Female gender					–	0.027
6. Age in years						–

*p < 0.05.

### Main Analyses

Results of the unconditional Model 1 displayed in Table 3 showed an ICC of 0.164 for Level 2 and 0.039 for Level 3, thus 16.4% of the variability of T2 individual autistic traits level was due to differences between raters and 3.9% due to differences between classrooms. Results of Model 2 indicated no significant effect of the level of autistic traits of the most liked peers at T1 on future individual autistic traits (*p* = 0.724), controlling for T1 individual level of autistic traits. Also, results of Model 3 indicated no significant effect of the level of autistic traits of the most liked peers at T1 on future individual autistic traits (*p* = 0.658), controlling for T1 individual level of autistic traits, general functioning, gender, and age. Hence, we rejected our first hypothesis. Considering the effects of the control variables, we found that more autistic traits at the beginning of the school year predicted more autistic traits at the end of the school year (*B* = 0.838, *SE* = 0.075, *p* < 0.001). In addition, a significant effect of T1 general functioning on the T2 level of autistic traits (*B* = −0.005, *SE* = 0.002, *p* = 0.017) was found, suggesting the greater general functioning a student showed at T1, the more autistic traits decreased. No main effects of gender (*p* = 0.868) or age (*p* = 0.970) were found.

**TABLE 3:** Prediction of individual autistic traits at T2 from T1 autistic traits of liked peers.

	Unconditional model 1 *B* (*SE*)	Model 2 *B* (SE)	Model 3 *B* (*SE*)	Model 4 *B* (*SE*)
**Fixed effects**				
Autistic traits of liked peers T1		0.021 (0.058)	0.027 (0.062)	0.280 (0.118)*
Individual autistic traits T1		0.856 (0.069)*	0.838 (0.075)*	0.834 (0.075)*
Level of general functioning T1			−0.005 (0.002)*	−0.006 (0.002)*
Gender			−0.006 (0.036)	0.111 (0.059)
Age			0.000 (0.005)	0.002 (0.005)
Autistic traits of liked peers T1 × Gender				−0.299 (0.134)*
**Variance components**				
Level 1 (within raters)	0.075 (0.010)*	0.029 (0.005)*	0.030 (0.005)*	0.028 (0.005)*
Level 2 (between raters)	0.018 (0.014)	0.012 (0.016)	0.007 (0.014)	0.007 (0.013)
Level 3 (between classrooms)	0.001 (0.011)	0.006 (0.016)	0.012 (0.014)	0.013 (0.013)
ICC 1 level 2	0.164	0.119	0.113	0.116
ICC 1 level 3	0.039	0.094	0.106	0.111

*p < 0.05.

To test our second hypothesis, which sought to determine whether gender moderates the effect of the level of autistic traits of the most liked peers on individual autistic traits, we added an interaction term for the level of autistic traits of the most liked peers and gender to the model (Table 3, Model 4). We found a significant interaction effect for gender (*B* = −0.299, *SE* = 0.134, *p* = 0.025). As the reference group was girls, this effect indicates that boys were less influenced by the level of autistic traits exhibited by the most liked peers than girls. We therefore accepted our second hypothesis that girls are more susceptible to peer influence on autistic traits than boys. Additional exploratory analyses indicated no significant moderation of the peer effect (as determined in Model 4) by students’ general functioning (*B* = 0.001, *SE* = 0.008, *p* = 0.886) and age (*B* = −0.024, *SE* = 0.020, *p* = 0.228).

To shed more light on gender differences regarding peer influence susceptibility, further interpretations can be made of the effects seen in Model 4. The significant simple effect of the most liked peers’ autistic traits level here refers to the group of girls (*B* = 0.280, *SE* = 0.118, *p* = 0.017). This effect indicates that girls were influenced by peers, even though there was no significant main effect in the overall sample (Jaccard and Turrisi, 2003). A one-unit increase in the level of autistic traits of the most liked peers at T1 coincided with an increase in girls’ individual level of autistic traits of 0.280 (on a scale from 0 to 2) over time. The size of the peer effect (standardized coefficient) was β = 0.262 and can be considered small according to Cohen (1988). Since there was no significant peer effect in the total sample (see Model 3), the significant simple effect in the group of girls in Model 4 in turn suggests that boys’ autistic traits level is not influenced by the peers’ level of autistic traits. This was confirmed by additional analyses considering only the subsample of target boys (*N* = 262). Using the same analysis procedure as in Model 3, we found no significant peer effect (*B* = 0.009, *SE* = 0.072, *p* = 0.904).

## Discussion

This study investigated peer influence on autistic traits and whether this differed by gender, focusing on students who attend special needs schools. Our results reveal that girls but not boys with ID and high levels of autistic traits are influenced by the autistic traits level of the peers they like most at school.

Results from our preliminary analyses should be considered to allow for better interpretation of our main findings. Generally, autistic traits decreased over the school year, which is in line with findings that the levels of autistic behavior among individuals with ASD can change over time, with a tendency to decrease from childhood to adolescence (Seltzer et al., 2004; McGovern and Sigman, 2005; Shattuck et al., 2007). Our analyses found less of a decrease for individuals with lower levels of general functioning, confirming earlier results showing less advantageous development of autistic traits in this group (Seltzer et al., 2004; Shattuck et al., 2007). When interpreting peer effects, it is also important to understand which students were preferred by students with high levels of autistic traits. According to teacher-reported peer nominations, target students preferred peers who were approximately the same age, and who exhibited lower overall levels of autistic traits than their own. This finding extends previous observations that individuals with an ASD diagnosis prefer to interact with people on the autism spectrum or with much younger or older individuals (American Psychiatric Association, 2013; Morrison et al., 2020). More than half of the nominated peers were male, mirroring the overall gender distribution among students attending the participating schools.

Our first hypothesis related to a general peer effect on autistic traits. While earlier research among typically developing children and adolescents found such effects for externalizing and internalizing behaviors (e.g., Erickson et al., 2000; Conway et al., 2011), we found no evidence of peer influence on autistic traits when considering the whole sample of boys and girls. This reduced susceptibility to peer influence in individuals with ID and high levels of autistic traits is consistent with studies showing that individuals with an ASD diagnosis and typical levels of intellectual functioning are less influenced by others (Izuma et al., 2011; Yafai et al., 2014; van Hoorn et al., 2017). Explanations may include general difficulties in social interaction and social communication, as well as problems in adaptive functioning among individuals with ID and high levels of autistic traits that impede the processes underlying peer influence. For example, difficulties perceiving and interpreting social cues from peers may result in decreased experience of social peer reinforcement in this group of students.

While there was no peer effect for the whole sample, testing our second hypothesis revealed the individual level of autistic traits of target girls was influenced by their preferred peers, a finding that did not hold for boys. This is in line with studies among typically developing students, which found susceptibility to peer influence differs between boys and girls, depending on the specific behavioral domain considered (Erickson et al., 2000; Stevens and Prinstein, 2005; Steinberg and Monahan, 2007; Parsai et al., 2009; van Zalk et al., 2010; Conway et al., 2011; Müller et al., 2016). An explanation regarding autistic traits may relate to findings that girls with an ASD diagnosis have closer social relationships (Head et al., 2014) and show more motivation for social contact than boys with ASD (Sedgewick et al., 2016). Higher social motivation for interaction with peers and a tendency for Camouflage may increase girls’ willingness to adapt or change their own behavior to fit in among peers. In the context of this study, this could mean that target girls adapted their autistic traits to the lower levels of autistic traits of their preferred peers. Such an adaption may in turn allow girls with high autistic traits to have more social contacts and therefore more opportunities for receiving peer influence. While these underlying processes could not be tested within the current study, they suggest directions for future research.

### Implications

The results from this study provide several theoretical and practical implications. Overall, this study shows that individual factors, such as earlier autistic traits level and general level of functioning, appear to play a more important role for autistic traits development in students with ID than peer influence. This finding is in contrast to other domains of behaviors, such as externalizing behavior among adolescents, where peer influence is considered a major explanatory factor (e.g., Warr, 2002). Given that ASD is a developmental disability and autistic traits are known to be influenced by various pre-, peri-, and neonatal biological factors (see, e.g., Ratajczak, 2011), this finding is perhaps not completely surprising. Nevertheless, it appears autistic traits development is likewise not completely unaffected by peer characteristics, at least not in girls with ID. This insight is new to our understanding of autistic traits development and is in support of the recent calls for attention to gender differences in ASD (Kirkovski et al., 2013; Hull et al., 2017). It could be that higher social skills are not only responsible for more peer influence susceptibility among girls with ID and high levels of autistic traits (see discussion above), but also the reverse: If these girls are more responsive to peer influence than boys, they may also benefit more from peer learning in the social domain and therefore develop greater social competence than the boys. Research on peer influence susceptibility among individuals with high levels of autistic traits may therefore also hold potential explanations for gender differences in ASD in the social domain (Head et al., 2014; Sedgewick et al., 2016; Dean et al., 2017). Although our study cannot test these assumptions, future research should systematically examine the directions and interplay between social skills and peer influence susceptibility in boys and girls with high levels of autistic traits.

In terms of practical support, our finding of peer influence susceptibility of girls suggests teachers might seek to create situations in which girls with ID and high levels of autistic traits can benefit from social learning among their peers (e.g., through social games or specific group work assignments; see also Farmer et al., 2018). Regarding optimal student group composition, studies have shown that behavioral heterogeneity within a group can be advantageous for social behavior development (Müller et al., 2021). Given a general susceptibility to peer influence on autistic traits, girls with ID and high levels of autistic traits may benefit most from interactions with students with low levels of autistic traits and high social competence, which can be considered when assigning students to groups.

While the present study did not identify peer influence susceptibility with regards to autistic traits in boys with ID, this does not imply this group is generally unresponsive to peer influence. For example, it remains to be determined whether boys’ autistic traits may benefit from peer influence when professional support is provided, for example directing a student to pay attention to certain socially competent peer behaviors. Also, boys with ID and high levels of autistic traits could be influenced by peers in other domains of learning and behavior, besides autistic traits. Overall, more research is needed to better understand the basic processes of peer influence on autistic traits and to improve the corresponding perspectives on support provision for individuals concerned.

### Limitations and Future Research Directions

To our knowledge, this study is the first to investigate the influence of autistic traits levels of preferred peers on individual autistic traits development in children and adolescents. By considering this question in a sample of students with ID and high levels of autistic traits this study provides insights on a population that tends to be at risk of less advantageous outcomes (e.g., less improvements in social reciprocity, greater deficits in social behavior; see, e.g., Shattuck et al., 2007; Matson and Shoemaker, 2009). Due to its longitudinal research design, comparably large sample size, and high participation rates in classrooms, we were able to gain reliable information on the questions examined.

Nevertheless, this study also had limitations. First, our data relied on school staff as our sole source of information. There are several reasons we used staff reports. Generally, staff members make daily observations of the students they work with, offering direct insight into the behaviors and peer relations among students at special needs schools. Accordingly, staff reports on the behaviors of children and adolescents with developmental disabilities, including autistic traits, are considered reliable and valid (e.g., Einfeld and Tonge, 1995; Goodman, 2001; Constantino and Gruber, 2005). From a pragmatic point of view, using staff reports made it possible to obtain continuous data on autistic traits levels for 1,125 students. However, in terms of assessments of peer relations certain questions must remain open. Investigating a student group who often cannot report on their peer relations themselves, we could not test in how far the staff reports matched what the students’ themselves would have reported. Also, we did not have detailed information on specific aspect of peer relations, such as how much time target students spent with their preferred peers, or what activities they did together. Furthermore, the exclusion of target students for whom no nominations were provided by staff may have influenced the results. As this group had lower levels of general functioning and exhibited more severe levels of autistic traits, the present study results tend to be most reliable for the more capable students within the population of students with ID in special needs schools. Future research may use more fine-grained analyses of social interactions among peers (e.g., using direct behavior observations) to allow further insights with a focus on profoundly handicapped students. To avoid the exclusion of specific student groups from samples, it would also be useful to additionally investigate the influence of involuntary peer groups on autistic traits, such as from all classmates.

Second, in this large-scale study using anonymous data collection we had no information on students’ clinical diagnoses of ASD. We therefore had to rely on a screening algorithm and considered autistic traits on a continuum. Related to this, the origin of autistic traits could not be clearly attributed to either ASD or ID. Future studies would therefore benefit from having more information on whether students have an ASD diagnosis based on extensive clinical assessments.

Third, the generalizability of our results on students with ID from special needs schools to students without ID and to other school contexts is limited. Given that in Switzerland about 25–73% (depending on the age) of children and adolescents with an ASD diagnosis attend special needs settings (Eckert, 2015), the focus of the present study well represents the social situation of many students with autistic traits in this country. However, even after controlling for earlier autistic traits and general functioning, our results do not allow for conclusions on how peer influence affects autistic traits development among those with ASD and typical levels of intellectual functioning. These students may use cognitive competences to compensate for certain social difficulties (e.g., Seltzer et al., 2004), which could also affect their peer influence susceptibility. In addition, this group of children and adolescents often attends regular school settings, which have a peer context that differs from the one in special needs schools. It will therefore be important to extend our findings by examining peer influence on autistic traits in students attending regular school settings.

A fourth limitation is that this study investigated peer influence with regard to only one behavioral aspect, namely the overall level of autistic traits. To follow up on findings by Nenniger and Müller (2020), future studies may also investigate peer influence regarding different subtypes of autistic traits, such as social communication, social interaction, and repetitive behaviors and interests.

In sum, for students with ID and high levels of autistic traits this study indicates that girls’ but not boys’ autistic traits development is influenced by the level of autistic traits among their preferred peers in special needs schools. Given that peer influence research on autistic traits is still in its infancy, we hope our findings will stimulate further research on the role of peers in the development of children and adolescents with high levels of autistic traits, potentially resulting in strategies to practically support these students in their peer context.

## Data Availability Statement

The raw data supporting the conclusions of this article will be made available by the authors, without undue reservation.

## Ethics Statement

The studies involving human participants were reviewed and approved by the Institutional Research Commission of the Department of Special Education of the University of Fribourg. Written informed consent from the participants’ legal guardian/next of kin was not required to participate in this study in accordance with the national legislation and the institutional requirements.

## Author Contributions

GN collected part of the data used, conducted all analyses, and wrote this manuscript. VH collected part of the data used and contributed to the analyses and to manuscript revision. CM was the principal investigator of the study which this project is part of, and contributed to conception and design of the study and to manuscript revision. All authors read and approved the submitted version.

## Conflict of Interest

The authors declare that the research was conducted in the absence of any commercial or financial relationships that could be construed as a potential conflict of interest.

## Publisher’s Note

All claims expressed in this article are solely those of the authors and do not necessarily represent those of their affiliated organizations, or those of the publisher, the editors and the reviewers. Any product that may be evaluated in this article, or claim that may be made by its manufacturer, is not guaranteed or endorsed by the publisher.
